# Defining the content of a website on advance care planning in dementia: a focus group study with family and health professionals

**DOI:** 10.1186/s12911-023-02359-1

**Published:** 2023-11-08

**Authors:** Charlèss Dupont, Tinne Smets, Fanny Monnet, Lara Pivodic, Aline De Vleminck, Chantal Van Audenhove, Lieve Van den Block

**Affiliations:** 1 VUB-UGhent End-of-Life Care Research Group, Vrije Universiteit Brussel (VUB), Brussels, 1090 Belgium; 2 Department of Family Medicine and Chronic Care, Vrije Universiteit Brussel (VUB), Brussels, 1090 Belgium; 3 LUCAS Center for Care Research and Consultancy, KU Leuven, Leuven, Belgium

**Keywords:** Advance care planning, Dementia, User-needs, Internet-based intervention

## Abstract

**Background:**

Advance care planning (ACP) is a process that enables individuals to define goals and preferences for their future care. It is particularly relevant for people with dementia and their family. Interactive tools, such as websites, that encourage reflection, communication and/or documentation, may support this group in the ACP process. However, considering the specific needs of people with dementia, it is important to develop adapted tools for this population. This study was conducted to define the content of an interactive website for people with dementia and their family caregivers to support them in ACP and to assess the barriers and facilitators for potential users in finding and using such a website from the perspective of family caregivers and healthcare professionals.

**Methods:**

Online focus groups with family caregivers (serving both as potential users and proxies for people with dementia) and healthcare professionals caring for people with dementia, using a semi-structured topic guide. To analyse the data, we used thematic framework analysis with a combination of deductive and inductive approaches to coding.

**Results:**

We conducted 4 focus groups with family caregivers of people with dementia (*n* = 18) and 3 with healthcare professionals (*n* = 17). Regarding the content of the website, participants highlighted that information on ACP (what and why) and guidance on how to start talking about ACP throughout the dementia trajectory should be included on the website. To increase the usability of the website, most participants considered a text-to-speech and a print option as important functionalities. A lack of computer literacy was found to be the most significant barrier to finding and using the website.

**Conclusion:**

A website for people with dementia and their family caregivers to support them in ACP should focus on comprehensive content on ACP, peer testimonials, and interactive communication tools. Moreover, there should be certain flexibility in navigating through the website so people with dementia and their family caregivers can use it at their own pace. As the next step, we will include people with dementia in developing the website.

## Introduction

Advance care planning (ACP) has been defined as a process that enables individuals to define goals and preferences for future care, to discuss these preferences with family and healthcare providers, and to record these preferences and choices [1, 2]. In recent literature the concept of ACP has been broadened from a clinician-led process that stresses the need to complete advance directives to an ongoing process of communication between patients, their family, and healthcare professionals [3, 4].

While ACP might be important for all patient groups, it has particular relevance in dementia. Because of the cognitive and functional decline in dementia and the disease’s difficult-to-predict course, it has been advocated that ACP should be initiated in the early stages of dementia. This allows the patient to be actively involved in decision-making about their future care [5]. Furthermore, when initiating ACP early on in dementia, people with dementia can be fully involved and family caregivers can gain a better understanding of the care and treatment preferences of the person with dementia and, hence, they may experience less doubt and stress when making proxy-decisions on behalf of the person with dementia [6, 7]. However, people with dementia and their family caregivers often indicate that they lack ACP knowledge and, although they want to, they cannot find the right time to start the conversation [8]. In the last few years, many new interventions have been developed to support people with dementia and their families in ACP. However, most of these interventions focus on ACP between the patient/family caregivers and healthcare professionals [9–12], while studies show that people with dementia and family caregivers also want to discuss future care and preferences in the family context—outside of the professional care settings [8, 13, 14].

To support such conversations, using interactive, web-based ACP support tools (e.g., websites, apps) can be beneficial (1). These web-based tools offer many advantages – including the possibility to access them at any preferred time and location [15] – and often contain interactive elements that allow the content to be tailored to the user’s needs [15]. This can be an advantage for people with dementia, as their abilities and the needs of their family caregivers change during the disease trajectory [5]. Web-based ACP tools can also facilitate the initiation of conversations, which has been identified as an important barrier in ACP [14, 16, 17]. However, despite their potential, the currently available web-based ACP support tools are not specifically developed for people with dementia and their family caregivers [18]. Considering the specific trajectory of the disease and the need to initiate ACP early [8, 19, 20], it is important to develop web-based tools that are adapted to the needs and preferences this population.

To ensure usability and future uptake of web-based tools, understanding people with dementia and their families’ needs and preferences is essential. This study is part of a larger study in which we developed an interactive, web-based ACP tool for people with dementia and their families [21]. As a first step, we conducted the present qualitative study to identify what should be included as content, the mode of delivery, and what may influence using and finding such an ACP support tool in this population. We have involved family caregivers acting as users and proxies for people with dementia, and professionals working with people with dementia. The specific aims of this study are to identify:

1. the content of the interactive ACP tool;
2. the delivery of the content (i.e., functionalities) of the interactive ACP tool;
3. the perceived barriers and facilitators for finding and using the interactive ACP tool.

## Methods

### Study design

**Fig. 1:**
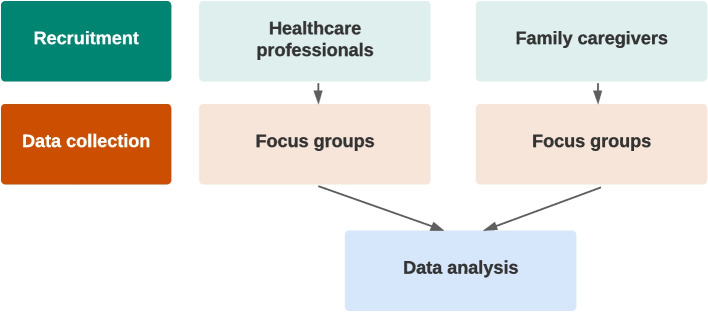
An overview of the study procedures

Recruitment and focus groups were conducted with family caregivers and healthcare professionals of people with dementia separately. For data analysis, the focus groups’ data were combined (Fig. 1). Focus groups were considered the most appropriate method for this study because they can help generate individual perspectives while also creating collective interaction. This interaction between participants often results in rich discussions and can lead to the emergence of diverse perspectives, shared experiences, and group dynamics (1). Due to the COVID-19 restrictions during data collection, the focus groups were held online. We limited the number of participants per focus group because it is recommended to have fewer participants (4–6 participants) in online focus groups to manage the discussion and facilitate interaction among the participants (1). Based on earlier qualitative research, we aimed to include 15 healthcare professionals and 15 family caregivers [22–25]. The findings of our study are reported following the Standards for Reporting Qualitative Research (SRQR) guideline [26]. Because the web-based ACP support tool will be the format of a website, we call it hereafter ‘interactive ACP website.

#### Participants and recruitment

Participants were recruited using 2 strategies: [1] Family caregivers were recruited via peer support groups for family members of persons with dementia in Flanders, also accommodated within the Alzheimer Liga Flanders; [2] Healthcare professionals were recruited via the researchers’ professional networks of individuals working in the Flemish dementia care field and the Expertise Centrum Dementia Flanders. Inclusion criteria (Table 1) were self-assessed (i.e., these inclusion criteria were mentioned in the recruitment call) and were checked by the researcher (CD) before sending the invitation to the participants.

**Table 1:** Participant inclusion criteria

Family caregivers	Healthcare professionals of people with dementia
Being a primary caregiver of a person diagnosed with dementia;	Professional caregiver in Flemish dementia care field;
Having participated (> 3 times) in one of the family groups dementia and/or early-onset dementia of the Alzheimer Liga Flanders;	Having frequent (> 4 times a week) interaction with people with dementia and their family caregivers;
Having access to the internet on the day of the focus group;	
Having a certain degree of computer-literacy and feeling comfortable discussing topics online;	
Having an interest in, and being willing to talk about, the topic of the focus groups;	
18 years of age and older;	
Fluent in Dutch	

A recruitment call was distributed by the researchers and the recruitment channels (i.e., Alzheimer Liga Flanders and the Expertise Centrum Dementia Flanders) via e-mail, newsletters, and social media. Family caregivers and healthcare professionals who were interested in participating were asked to express their interest in an e-mail to the researcher (CD). Interested participants received an information letter and an online informed consent form by e-mail and were asked to sign the online form using (the Open Source software) Limesurvey.

#### Data collection

All participants who gave consent received, directly from the researcher, a personal meeting link to participate in an online focus group. Each online focus group was led by an experienced moderator (CD or LVdB) and an observer (CD, TS, or FM), who also assisted when technical problems occurred, moderated the chat, and took notes. Family caregivers were asked to respond from their own perspective as a caregiver as well as from that of their relative with dementia. We asked healthcare professionals to give us their views on what they thought would be important for people with dementia and their family.

The focus groups were conducted using a semi-structured topic guide and PowerPoint with possible relevant content topics for the ACP website to guide the discussion. The following topics were shown to the participants during the online meeting: [1] information on ACP, [2] information on legal frameworks, [3] reflecting on readiness for and timing of ACP, [4] reflecting on personal values and goals, [5] reflecting on preferences regarding future care, [6] reflecting on uncertainties and consequences, [7] reflecting on preferences regarding last days of life, [8] reflecting about a proxy decision-maker, [9] appointing a proxy decision-maker, [10] communication with family, [11] communication with healthcare professionals, [12] and documentation of ACP [1, 2]. Before showing these topics to the participants, we explained and showed them the broad definition of ACP by Rietjens et al. [1]. Preferences regarding functionalities – how the content is delivered (e.g., video), options for a larger font, text-to-speech option, etc. – and the possible barriers and facilitators to finding and using the website were assessed using open questions. Moreover, we also asked about the need for separate sections within the ACP website for people with dementia, family caregivers and dyads.

Each focus group was scheduled for 1.5 h. If this timeframe was not enough, we sent the questions about the barriers and facilitators for using and finding the interactive ACP website via e-mail. The focus groups were held between January and April 2021.

#### Data analysis

The focus groups were recorded and transcribed verbatim. All transcripts were pseudonymised by the researcher (CD), who was involved in all focus groups. To analyse the data, we used framework analysis (65). The framework analysis approach in thematic analysis involves several stages, including data familiarisation, thematic framework development, indexing all study data against the framework, charting to summarise this data, and lastly mapping and interpretation (65). First, two researchers (CD and FM) coded 30% of the transcripts in NVivo (version 1.4.1) using both deductive and inductive approaches to qualitative data analysis. We started with predetermined codes and analysed the transcripts to find excerpts that fit these codes (deductive approach). For the data that did not fit pre-determined codes, we created new codes (indicative approach). The coding tree with codes for the deductive approach was built on the content topics presented during the focus groups (i.e., a content topic was a theme). Participants’ responses to questions about the barriers and facilitators to finding and using the ACP website received via e-mail were added to the transcript of the focus group and analysed simultaneously. The identified themes were reviewed with the researchers involved and consensus was sought to build the framework. After, all transcripts were analysed using the build framework. Finally, the results were ordered and discussed with all authors of this article for interpretation.

#### Ethics

The study was carried out in accordance with relevant guidelines and regulations and received Ethics approval via the Ethical Review Board of UZ Brussels, Belgium (BUN: 1432020000277).

## Results

We performed 4 focus groups with family caregivers (*n* = 18); 10 persons were family caregivers for their partner and 8 for their parent(s). Most of the family caregivers were female (*n* = 14). We conducted 3 focus groups with healthcare professionals (*n* = 17 (of which *n* = 11 were female); occupational therapist *n* = 4, physician *n* = 3, reference person dementia *n* = 3, employee of dementia organization *n* = 3, nurse *n* = 2, speech therapist *n* = 1, and director of nursing home *n* = 1). Afterwards, participants of 3 focus groups (1 with family caregivers and 2 with healthcare professionals) received (via e-mail) the questions about the barriers and facilitators to finding and using the ACP website.

After the first focus group, we slightly adjusted the focus group guide and PowerPoint slides by adding 3 topics that the participants mentioned as important topics: [13] What if my loved one does not communicate (much), [14] what if there are difficult end-of-life decisions to be made, and [15] what if there is a disagreement in the family. We deleted the division within the ACP website of possible target groups (i.e., people with dementia, family caregivers, and the dyad).

### Needs and preferences for the content of the ACP website

Both family caregivers and healthcare professionals addressed comparable content needs. They considered content on information and guidance in ACP as important. Based on our findings, we identified 3 main topics regarding the content of the website:

1. Information about Advance Care Planning

Most participants valued the topics ‘information on ACP’ and ‘legal frameworks’ for the content of the website. Especially information on ‘what is ACP’ and ‘why should one perform ACP’ was deemed as important to be included. Several participants stressed the importance of straightforward and realistic information on ACP, including a precise but clear explanation of existing ACP legal frameworks. Additionally, most participants thought the information on ACP on the website should be adjusted to the trajectory of dementia. Family caregivers, in particular, indicated that they think it is important that the information on the website take the declining cognitive capacities in the different stages of dementia into consideration and should include tips on how to do ACP throughout the trajectory.

Family caregiver 3 (partner): *“It is important to actually look at it in terms of the stage of the dementia, that it is very important to build on that. People with dementia who are still able to start those conversations themselves or indicate to the family caregiver that they want to talk about it – so the stage of dementia, and how the person with dementia deals with it, all play a role in the story of ACP."*

Also, according to most participants, ACP readiness can differ within a dyad, and this should be mentioned on the website. For example, a family caregiver could be ready to discuss possibilities for future care, while the person with dementia only wants to discuss her/his preferences around social care. This should be clear in the information about what ACP is, when and how to conduct ACP, what possible barriers to expect, and how to deal with them. Lastly, most participants agreed that the additional topics added after the first focus group – i.e., what if my loved one does not communicate (much), what if there are difficult end-of-life decisions to be made, and what if there is a disagreement in the family – are very relevant and should be mentioned on the website.

2. Guidance on how to start and conduct an ACP conversation:

Most participants mentioned that they found talking about ACP – and, more specifically, starting an ACP conversation – to be the most difficult. Especially family caregivers said they had difficulties bringing up the topic, both with the person with dementia, and with a healthcare professional. Most participants indicated that they would like to see content on the website about *‘how do I start?’* and preferably with an interactive aid that can function as a conversation starter:

Family caregiver 2 (parent has dementia): *"You can find a lot of information about advance directives and what it is, but how do you start it [ACP], which tool can you use? That's something that is not easy to find, or you have to ask services or organizations about it, but I haven't found many tools to support us, and that's something that can be very useful.”*

Moreover, most healthcare professionals and family caregivers said that it would be important to distinguish between different kinds of family caregivers. For example, a family caregiver who is a child may have different needs regarding ACP communication than a family caregiver who is a partner, as their relationship with the person with dementia is different. Most family caregivers also stressed that, when they would be able to talk about ACP among each other (person with dementia and the family caregiver), it would be easier to discuss ACP with a healthcare professional:

Family caregiver 6 (parent): *“I think it should mostly be done between the family and maybe they (i.e., the person with dementia and their family) will start talking to their caregiver more easily."*

3. Information about the disease and dementia care

Although family caregivers found information about dementia, its prognosis, and possibilities for (future) care important, they did not necessarily want it to be available on the ACP website. They are aware that such information is already available on the internet, though they thought that it would be important to refer to other websites about dementia for specific information on the disease and care because the information is, according to them, needed to be able to discuss ACP. Several healthcare professionals also suggested referrals to other websites on dementia (e.g., hyperlinks) because it is important to know more about the trajectory of dementia when conducting ACP.

### Needs and preferences for delivery of the content

All participants were very clear about how the content should be delivered. The content on the website should be easy to understand, convenient to look up, and relatable. Participants highlighted several ways to deliver the content, which we categorized in 4 ways:

- Presenting ACP as a process: According to the participants, the ACP website should consider the need for flexibility and should be tailored to the different needs of persons regarding the timing and their readiness to engage in ACP. Therefore, it should be possible to use the ACP website in every phase of readiness – i.e., users can start and quit when and wherever they want. Family caregiver 7 (partner): “*I think showing that it is a process is very important. You can't do that [ACP] all at once, it only hits you in pieces – and now after [I have done] everything, I can see it [ACP] in its entirety, whereas at the time I couldn't see it that way.”*
- A section with frequently asked questions (FAQ): Participants would like to see a section where they can easily search and find information that they are looking for. According to family caregivers and the healthcare professionals, people often have the same kinds of questions, and a place for them to easily search and find what they need would be convenient.
- A glossary: All participants valued an overview and explanation of important ACP terms, as this is, according to the participants, not easy to find on the internet.
- Testimonials: Participants considered testimonials and peer support to be helpful content in supporting ACP. They would value stories on experiences about how others conducted ACP, what they struggled with, and what helped them to start an ACP conversation.

Family caregiver 16 (partner): *"It would help when you hear others about how they conducted ACP; what did they experience with their partner who did not accept their diagnosis, and what did they do so they were able to talk anyway."*

Physician 2: "You can also use testimonials of peers so people can see others also find it difficult."

For their part, healthcare professionals mentioned that testimonials about how they also sometimes struggle with starting an ACP conversation with their patients could lower the threshold for patients and family caregivers to start an ACP conversation with the healthcare professional.

### Needs and preferences for the functionalities of the ACP website

Family caregivers and healthcare professionals addressed similar needs and preferences with regard to the use of functionalities on the ACP website. According to participants, when adding functionalities to the ACP website, they should benefit usability (i.e., they should support the user in easily using the ACP website). Four functionalities were mentioned that might facilitate the ease of use of the ACP website:

- Provide clear navigation: the navigation of the ACP website should be clear, so that it is easy for the user to navigate and find the content they are searching for. For example, the navigation in the ACP website should always be visible, and it should be easy to return to the homepage.
- Provide a print option: on every page of the ACP website, a print option should be available so that the user can print the content and read it on paper.
- Use a text-to-speech option: just like the print option, on every page there should be an option to transform the text into speech. This way, people who have reading problems can visit and use (listen to) the ACP website.
- Provide an option to increase the font size: it should be possible to increase the font size of every paragraph individually. This way, people can read the part they want without everything in the whole ACP website getting bigger.

### Barriers and facilitators to finding and using the ACP website

#### Barriers to, and facilitators for, finding the ACP website

According to healthcare professionals and family caregivers, the biggest barrier will be that people most probably will not find the ACP website ‘spontaneously’ and they will need some kind of introduction via an organization or healthcare professional. Also, because there is still a large taboo concerning ACP, and people think it is only about dying, they may not search for the ACP website on their own.

Participants suggested promoting the ACP website via dementia organizations, insurance funds and healthcare professionals. This would help people find the ACP website, as these parties could refer people with dementia and family caregivers to the ACP website. Participants also mentioned using a variety of media to promote the ACP website, including: social media, other websites, videos in healthcare professionals’ waiting rooms, and the use of print media like brochures. Especially in healthcare settings like hospitals, brochures can be useful because people often spend time in a waiting room.

#### Barriers facilitators to using the ACP website

According to the participants, the most important barrier to using the ACP website is that people with dementia, especially those with late-onset dementia, but also their family caregivers, may lack the necessary computer skills. Moreover, many people with dementia have reduced ability for abstract thinking. Healthcare professional 8 (nurse): *“Digital literacy can be a problem in addition to impaired abstract thinking skills.”* However, participants did believe the population of people with dementia and family caregivers is changing, since the use of computers and tablets have become far more widely used in recent years. Many people with young-onset dementia, in particular, have been working most of their life with a computer, and this will soon also apply for people with late-onset dementia.

Furthermore, according to some participants, the ACP website would be difficult for the person with dementia to use by her/himself because of their deteriorating cognitive abilities. However, the use of functionalities to adjust the delivery of the content (i.e., text-to-speech, larger font size) could support people in the earlier stages of dementia when using the ACP website.

Another facilitator, according to participants, is the possibility of using the ACP website at the person’s own pace in terms of timing and readiness. Family caregiver 3 (partner): "*At that time, we did not get any help from anyone to talk about it [ACP] and I found it difficult to start talking about it with the two of us. At one point it was vaguely mentioned, but not in detail. And indeed, it is a very good way to start a conversation and maybe it is also necessary to let it rest for a while and say I'll come back to it. Yes, I think that is important to give the person with dementia some space and time to prepare.”*

A possibility for using the ACP website and returning to the same page (saving the user time) is a login system. Participants thought this would be interesting because it would give users the opportunity to stop and return when they want. Nevertheless, the use of a login system would be a big barrier, because users would have to their login codes.

## Discussion

Participants in this study considered information on ACP – including legal frameworks and guidance on thinking and talking about ACP – to be important content for the interactive website to support ACP. To support the accessibility and usability of the website, participants recommended the use of a text-to-speech option, a print option, and the possibility of increasing the font size. Participants suggested that healthcare professionals should be involved in guiding people with dementia and their family caregiver to the website since people may not find the website on their own.

Family caregivers and healthcare professionals addressed the importance of the content of ACP information, which should go further than explanations of advanced directives, and should provide guidance on how to initiate an ACP. They also considered it to be important to refer to other websites for information on dementia. Family caregivers need for information on ACP, dementia and the expected disease trajectory was also mentioned in Van Rickstal et al. (2019) exploratory interview study among people with young-onset dementia [27]. Providing information on ACP and dementia is crucial in assisting people with dementia and their family in ACP [28]. It could support them in actually starting to think about ACP [29, 30]. Moreover, the participants in our study suggested that the inclusion on the website of an interactive conversation starter would be useful in supporting people with dementia and their family caregivers to start talking about ACP. The use of interactive conversation starters to support discussions between a person with dementia and their family caregivers has already been used successfully in other domains (such as reminiscence therapy) and might stimulate meaningful conversations [31, 32].

Moreover, most participants in this focus group study stressed that whether or not someone engages in ACP depends on their readiness to think and talk about these difficult topics. Some people may not be ready to start an ACP discussion, and this readiness to engage in ACP can differ between the person with dementia and their family caregiver. The various needs in the timing of ACP – where some people with dementia only want to focus on day-to-day challenges and others want to start planning as soon as possible – is also shown in earlier studies [8, 20, 33, 34]. Therefore, information on the website should emphasise the possible differences in readiness, and the structure of the ACP website should allow users to access all parts of the website without having to follow a predefined chronological structure. This means that the users should have certain flexibility in navigating through the ACP website and should be able to use it at their own pace. However, existing ACP tools often use a predetermined path or step structure to go through the tool [18]. Although these existing ACP tools use a login system to try to provide flexibility by offering the option to ‘leave and return’ [18], participants in this study did not recommend using this functionality as it was deemed too difficult to use. These findings again show the importance of considering the end-user when developing new web-based tools.

Most of the participants in our study were convinced that functionalities should only be used if they benefit the accessibility and usability of the website. Three important functionalities were mentioned: a text-to-speech option, a print option, and the possibility of increasing the font size. Two of these functionalities (the print option and the option to increase the font size) are also recommended by the Alzheimer Association as important functionalities to consider when developing technology for people with dementia [35]. However, the use of text-to-speech is relatively uncommon on ACP websites. A recent systematic review that identified 30 ACP support tools that are publicly available for anyone to use and found that only 3 used a text-to-speech option [18]. However, using text-to-speech could increase the accessibility of a website for people with cognitive disabilities, as content can be presented in multiple modalities and be altered to the needs of the user [36].

Lastly, many participants indicated that people with dementia and family caregivers who want to conduct ACP conversations might, in many cases, not search for the website [8, 13, 14]. For this reason, healthcare professionals organisations should be stimulated to guide people to the website during consultations or via various media. This finding aligns with the known barriers for the initiation of ACP in dementia [37].

## Strengths and limitations

This study contributes to the limited research on the needs of people with dementia and their family caregivers regarding ACP [27], and this study is the first to assess the needs and preferences regarding a website to support people with dementia and their family caregiver(s) in ACP. In this focus group study, we included family caregivers of various ages and with varying relationships to the person with dementia and healthcare professionals from different disciplines involved in dementia care. We also evaluated barriers to, and facilitators for, finding and using the ACP website to anticipate possible barriers to implementation. From previous research, we know implementation of web-based tools in ageing populations is difficult [38]; and a recent systematic review of implementation of eHealth interventions for informal caregivers of people with dementia showed again the importance of thinking ahead about implementation in the real world [39].

This study also has some limitations. Because we did not have a prototype of the website yet and this study was a first abstract exploration of possible needs to support ACP via a web-based tool in a family context, we did not include people with dementia [21]. Our aim was first to get a general impression of the possible contents of the website to develop a prototype after. This is also recommended in literature on technology developed for people with dementia [21]. We included people with dementia in the development of the website prototype, and we used their valuable feedback to adapt content and functionalities of the website [21]. For this developed we utilised an agile development approach, including a user-centred design [21]. This way, the input of people with dementia and their family caregivers was central in the developed process of the website. Second, although we considered focus groups the most appropriate method for this study, the fact that we had to conduct them online may have influenced the interaction between participants.

## Conclusion

This study provides valuable insights from family caregivers and healthcare professionals regarding the content of an interactive website for people with dementia and their family caregivers, its delivery, potential barriers and facilitators for findings, and the use of the website to support ACP. Participants stressed the importance of comprehensive ACP information, testimonials, and interactive conversation starters. Flexibility in navigating through the website was deemed crucial so users could use the website at their own pace. Moreover, healthcare professionals are important in guiding potential users to the website. While the study lacked direct input from people with dementia, their perspectives will be taken into account in the development phase of the website. In conclusion, this study provides a framework for an ACP website tailored to the needs of people with dementia and caregivers.

## Data Availability

The datasets used and/or analysed during the current study are available from the corresponding author on reasonable request.

## Abbreviation

ACP: Advance care planning
